# The Mineralization of Various 3D-Printed PCL Composites

**DOI:** 10.3390/jfb13040238

**Published:** 2022-11-11

**Authors:** Artem Egorov, Bianca Riedel, Johannes Vinke, Hagen Schmal, Ralf Thomann, Yi Thomann, Michael Seidenstuecker

**Affiliations:** 1 G.E.R.N. Center of Tissue Replacement, Regeneration & Neogenesis, Department of Orthopedics and Trauma Surgery, Medical Center–Albert-Ludwigs-University of Freiburg, Faculty of Medicine, Albert-Ludwigs-University of Freiburg, Hugstetter Straße 55, 79106 Freiburg, Germany; 2 Institute for Applied Biomechanics, Offenburg University, Badstraße 24, 77652 Offenburg, Germany; 3 Freiburg Center for Interactive Materials and Bioinspired Technologies (FIT), Albert-Ludwigs-University Freiburg, Georges-Koehler-Allee 105, 79110 Freiburg, Germany

**Keywords:** PCL scaffolds, 3D printing, collagen coating, hydroxyapatite, alkaline phosphatase, poly-L aspartic acid

## Abstract

In this project, different calcification methods for collagen and collagen coatings were compared in terms of their applicability for 3D printing and production of collagen-coated scaffolds. For this purpose, scaffolds were printed from polycaprolactone PCL using the EnvisionTec 3D Bioplotter and then coated with collagen. Four different coating methods were then applied: hydroxyapatite (HA) powder directly in the collagen coating, incubation in 10× SBF, coating with alkaline phosphatase (ALP), and coating with poly-L-aspartic acid. The results were compared by ESEM, µCT, TEM, and EDX. HA directly in the collagen solution resulted in a pH change and thus an increase in viscosity, leading to clumping on the scaffolds. As a function of incubation time in 10× SBF as well as in ALP, HA layer thickness increased, while no coating on the collagen layer was apparently observed with poly-L-aspartic acid. Only ultrathin sections and TEM with SuperEDX detected nano crystalline HA in the collagen layer. Exclusively the incubation in poly-L-aspartic acid led to HA crystals within the collagen coating compared to all other methods where the HA layers formed in different forms only at the collagen layer.

## 1. Introduction

Demographic change is currently a much-discussed phenomenon whose consequences appear more clearly every year [1,2]. In Germany, every second person is 45 years and one in five is older than 66 years of age [3]. In the EU, the average age of the population continues to rise and one in five is already older than the age of 65 [4]. A well-known and well-followed consequence is the increase of musculoskeletal diseases and the related increase of clinical interventions on the musculoskeletal system. For example, the use of an endoprosthesis in the hip is the sixth most frequent operation in Germany [5]. Thus, the need for clinically proven and effective bone graft substitutes is also increasing. However, there are still known and documented problems with the use of metal implants. Among other things, the unequal relationship of elastic moduli between the metals used and human bone can lead to undesirable or too weak bone growth (so-called stress shielding) [6]. In addition, a metallic implant is usually installed in the body for a long period of time. Depending on the age of the patient at initial implantation, re-implantation of an endoprosthesis may be required after 10–15 years (knee), 15–20 years (hip), or 10 years (shoulder), depending on the site of implantation [7,8]. Such reoperations pose enormous risks to the health of patients, especially at advanced ages. Bone substitutes made of biodegradable biomaterials represent an innovative alternative, which ideally are dissolved by the body after preservation of mechanical stability and healing of the bone [9]. One such biodegradable biomaterial is polycaprolactone (PCL) [10]. It is a biodegradable semi-crystalline polymer. Its good formability at relatively low temperatures makes it ideal for additive manufacturing processes in the context of medical use [9]. Additive manufacturing processes offer enormous potential for individual patient care. If, for example, bone is missing after a fracture, this individual lesion can be converted into a digital three-dimensional construct using clinical imaging techniques (CT, MRI). In a further step, this three-dimensional (3D) model of the damaged bone can be used to develop a bone substitute that is perfectly adapted to the individual case. This should ensure mechanical stability and thus take over the function of the damaged bone. This individual replacement piece made of PCL also has the advantage that, as a biodegradable polymer, it can be degraded by the body once it has fulfilled its task and as the bone heals. This eliminates the need for a second surgery to remove the structure, with all the associated risks. To enable even faster recovery and new bone formation, autologous bone-forming cells can be applied to bone substitutes made of PCL (further scaffolds). These cells accelerate new bone formation within the printed scaffolds. Thus, the printed PCL scaffold serves as a supporting and guiding structure during bone regeneration. Moreover, the surface of the printed PCL scaffolds plays an important role in regeneration. This surface has hydrophobic properties and thus inhibits cell colonization. Cell adhesion and further cell profiling does not occur with pure PCL [10]. To improve cell adhesion and cell profiling, the printed scaffolds should provide an “in vivo”-like surface. This should be based on the microstructure of bone, with main components consisting of collagen and HA. In theory, coating with collagen has improved cell adhesion [11] and cell profiling. The formation of HA on the collagen layer provides further improvement of cell profiling [12]. The integration of HA would provide a biomimetic layer [13]. In the present study, different ways of preparing this collagen HA surface layer were applied and investigated. Thus, various methods for generating HA layers on or within the collagen coatings of 3D-printed PCL scaffolds have been investigated as a possible use as bone substitutes. Ordered nano-crystalline HA layers within the collagen are already one step closer to the generation of bone tissue in the petri dish.

## 2. Materials and Methods

### 2.1. Materials

Ethanol, magnesium-chloride-hexahydrate, and PCL (Mn = 45,000; Art. No. 704105) were purchased by Merck (Merck KGaA, Darmstadt, Germany). Hydroxyapatite (Art. No 677418), 1-ethyl-3-(3-dimethylaminopopyl)-carbodiimid hydrochloride (Art. No. SLBZ0862), potassium chloride (Art. No P5405), Trizma base (Art. No. T6065), magnesium sulphate (Art. No. M2643-100GM) and Alkaline phosphatase (Art. No. P7923-2KU) were purchased by Sigma-Aldrich (Sigma-Aldrich, St. Louis, MO, USA). Collagen I (Art. No. 354236) was purchased by Corning (Corning, NY, USA). Calciumchloride (Art. No. CN93.2), Sodium-di-hydrogenphosphate (Art. No. K300.1), sodiumhydrogencarbonate (Art. No. 6885.2), Sodiumhydroxide (Art. No. 6771.1), di-sodium-hydrogenphosphate (Art. No. T876.1), Sodiumdihydrogenphosphate monohydrate (Art. No. K300.1), Sodiumchloride (Art. No. HN00.2), and di-ammoniumhydrogenphosphate (Art. No. P736.1) were purchased by Carl Roth (Carl Roth GmbH, Karlsruhe, Germany). Poly-L-aspartic acid sodium salt was purchased by Alamanda Polymers (Alamanda Polymers Inc., Huntsville, AL, USA). Beta-glycerphosphate di-sodium salt pentahydrate was purchased by EMD Millipore (EMD Millipore Corp., Billerica, MA, USA). Goat anti mouse Alexa 488 antibody (Art. No: A-11001) was purchased by ThermoFisher Scientific (Thermo Fisher Scientific Inc., Waltham, MA, USA).

### 2.2. Methods

#### 2.2.1. 3D Printing of PCL Scaffolds

The PCL scaffolds were manufactured by using a 3D-BioPlotter (EnvisionTEC, Gladbeck, Germany) according to our previous work [11,14]. For this purpose, a 3D model of the scaffold was created in Autodesk Inventor (Autodesk Inventor 2019; Autodesk, San Raphael, CA, USA). This was assigned to the printer in the subsequent step. The 3D printing parameters used are shown in Table 1. Before 3D printing, the PCL used was dried for one hour in a desiccator under vacuum in silica gel. It was then transferred inside a cartridge into the print head of the 3D-BioPlotter, which had been preheated to 80 °C. After one hour, the PCL became liquid and thus ready for 3D printing. A glass plate, previously cleaned with isopropanol, was placed underneath as a printing surface. The 3D print was made by plotting 12 layers. Each layer contained a frame and an inner structure, the so-called base pattern, with a layer height of 0.17 mm. Thus, with 12 layers, the total height was 2.04 mm. This resulted in a cuboid scaffold. The dimensions of a scaffold were 8.4 mm × 8.4 mm × 2.04 mm.

The finished scaffolds were visually inspected for print defects and contamination, with discarding of defective specimens and storage of acceptable specimens at room temperature.

#### 2.2.2. Collagen Coating

The final printed pre-sorted scaffolds were dried and simultaneously sterilized by five immersions in an ascending alcohol series (30/50/70/80/96 and 100% ethanol) before coating with collagen. After drying the scaffolds, their surfaces were activated by plasma (Piezobrush PZ3, Relyon Plasma GmbH, Regensburg, Germany). For surface activation, the scaffolds were plasma treated with the Piezobrush 3 at 80% power according to the manufacturer instructions at room temperature in air. Each scaffold was always treated for 30 s, cooled for 10 s, and then treated again for 20 s. Immediately after activation, the activated scaffolds were transferred to a 24-well plate containing 600 µL of collagen solution per well. The well plate (with scaffolds and collagen) was then incubated on a moving plate at 4 °C for 72 h. The scaffolds were then removed from the collagen solution and dried at 37 °C for 24 h. To cross-link the collagen layer, the dried scaffolds were transferred to a cross-linking solution in the subsequent step. 10 mL of cross-linking solution consisted of 10 mL of 95% ethanol containing 95.85 mg of 1-ethyl-3-(3-dimethylaminopropyl)carbodiimide hydrochloride (EDC) powder. The solution was shaken using a shaker (IKA Shaker MS 3 Basic) until the EDC powder was completely dissolved. Then, the cross-linking solution was added to the dried scaffolds at 1 mL per well (thus also 1 mL per scaffold) and incubated for 16 h. After the incubation period, the final scaffolds were cleaned five times with deionized water and then five times with 70% ethanol and dried on a filter paper. For stable storage, the dry scaffolds were stored in a new corrugated plate at room temperature.

#### 2.2.3. Methods for Inserting Hydroxyapatite

For the following experiments, at least 10 scaffolds were coated with the different methods. All experiments were repeated at least three times.

##### Collagen-Hydroxyapatite Coating

The procedure was similar to the collagen coating process presented in the previous chapter. First, 5% by weight of HA was added to the collagen as a nanopowder. The collagen-HA solution was further placed in an ultrasonic bath (Elmasonic P 60H, Elma Schmidbauer GmbH, Singen, Germany) at a frequency of 120 Hz for 1 h before addition to prevent rapid agglomeration. During the ultrasonic bath, the rising temperature of the water was steadily cooled down to 20–25 °C with double distilled water ice to prevent denaturation of the collagen. Since the addition of HA leads to gel formation [15] in the type of collagen used (Corning), caused by the basic effect of HA, 0.02 molar acetic acid was added. The acetic acid corresponded to the solvent of the Corning collagen solution. Furthermore, the amount of HA was reduced to 2% by weight so that the gel could be liquefied again. The liquid collagen-HA solution was again shaken with a Minivortex (Roth) to prevent agglomeration of the HA. In the subsequent step, 600 µL of this collagen-HA solution per well was added to the scaffolds. The following steps were identical to those for collagen coating. With 5% *w*/*v* HA, collagen gel formation occurred. Only when using 2% *w*/*v* HA nanopowder in the collagen and diluting with 0.02 M acetic acid could gel formation be prevented.

##### Immersion in SBF

Based on the findings of Poh et al. [16] and Vaquette et al. [17] immersion of objects in simulated body fluid (further referred to as SBF) leads to the formation of an HA layer on the surface of the inserted objects. According to Gomori et al. [18], this results from the well-known reaction of phosphates in calcium-rich environments. Both are sufficiently present in SBF, above the amounts required for the formation of HA. Furthermore, the formation time of the HA layer is shortened by increasing the ion concentration [16]. This was used in this variant for application for collagen coated scaffolds by increasing the ion concentration tenfold. First, 10× SBF was prepared according to the formulation of Yang et al. [19]. For this purpose, the substances shown in Table 2 were added sequentially in the order and amount listed until they dissolved completely in 1 L double distilled water. To do this, the sequence must be followed and the substances must be completely dissolved before the next substance is added to avoid precipitation of the dissolved substances. The final 10× SBF solution can be stored at 4 °C for several weeks without precipitation of the dissolved substances.

Before storing the coated scaffolds in 10× SBF, the pH of the 10× SBF solution was adjusted to pH 6 with NaHCO_3_. Then, 1 mL of the 10× SBF was added per scaffold to a 24-well plate loaded with scaffolds (including collagen coating) in the subsequent step. The well plate was then transferred to a drying oven at 37 °C and the well was gently shaken after 15 min. After 30 min, the medium was renewed by pipetting off the old solution and pipetting in fresh 10× SBF with pH 6. This step was continued throughout the duration of the experiment. To study different film thicknesses, different samples were stored in 10× SBF for 1 h (10× SBF 1 h), 2 h (10× SBF 2 h), 4 h (10× SBF 4 h), and 8 h (10× SBF 8 h), respectively. Then, 30 min after the last change of medium, the samples were transferred to 0.5 M NaOH for 30 min. This step homogenizes the resulting HA layer. After the time elapsed, the final samples were washed with double distilled water until the pH value of the water being washed out reached approximately pH 7.

##### Surface Coating with Hydroxyapatite by Addition of ALP

According to Jaroscewicz et al. [20], it is possible to produce an HA layer with the help of ALP. ALP present in the body catalyzes the hydrolysis of phosphate monoesters. Starting materials of these reactions are inorganic phosphate and an alcohol [18]. In an environment enriched with the necessary amount of calcium, a reaction of the free calcium and the inorganic phosphate now occurs. This leads to the formation and precipitation of HA. This reaction was already observed by Gomori [18] in 1953. For its preparation, a solution enriched with the necessary concentrations of substances had to be prepared. The composition of the solution called “phosphatase incubation medium” (further referred to as PIM) was prepared as listed in Table 3 according to the work of Jaroszewicz et al. [20]. A total of 45 mL of PIM was obtained. The indicated substances were added one by one to double distilled water in the indicated order, using a magnetic stirrer. The next substance in the sequence was added only when the previous substance had completely dissolved. A measure of 45 mL of double distilled water was used as solvent (see Table 3).

Freshly collagen-coated scaffolds were prepared for addition by storage in 1 mL 1× PBS/scaffold. The prepared 45 mL PIM were mixed with ALP. For this purpose, 10 nL of ALP per scaffold should be added to the PIM just before incubation. To achieve this small amount of ALP, the ALP was diluted with PIM to the extent that 10 nL of ALP is effectively dissolved in one milliliter. After adding the ALP to the PIM, 1 mL per scaffold of the prepared solution was immediately added to a 24 well plate loaded with scaffolds. The well plate was then transferred to a slow-moving moving plate at 37 °C in a drying oven. Here, the scaffolds remained for 1, 3, and 6 days, respectively, without changing the medium. After the scaffolds were removed, they were washed three times for 15 min in double-distilled water on a moving plate in a drying oven at 37 °C and then air-dried on filter paper. 

##### Mineralization of Collagen with Poly-L-Aspartic Acid

Based on the work of Deshpande et al. [21], it is shown that the presence of polyaspartic acid (further polyASP) leads to biomineralization of the collagen itself. Using this approach, four initial solutions were prepared for the generation of mineralization of collagen. For the first solution, 10× phosphate buffered saline (further referred to as 10× PBS) was prepared following the sequence listed in Table 2. This was then diluted to 3.4× PBS. For the second and third starting solution solutions, a 6.8 mM CaCl_2_ and 4 mM (NH_4_)_2_HPO_4_ solutions were prepared. Poly-L-aspartic acid as the fourth starting solution was brought to a 500 µg/mL solution by dissolving and diluting from a 5 mg/mL solution. These prepared starting solutions were mixed in equal parts (25 µL each) to make a total of 100 µL of mineralization solution. Then, 20 µL of the mineralization solution per scaffold was pipetted as a drop on a Petri dish with a diameter of 5 cm and 5 scaffolds each. Here, the drop was added to the center of the scaffold. The Petri dish was placed inside a home-made humidity chamber. The humidity chambers were sealed and placed in the drying oven (Memmert UM200, Memmert GmbH & Co KG; Schwabach, Germany) for incubation at 37 °C for 6 h. At the end of the 6 h, the finished scaffolds were briefly washed with double distilled water warmed to 37 °C and transferred to filter paper for drying.

#### 2.2.4. Characterization of the Scaffolds and Coatings

##### Characterization by 3D Laser Scanning Microscopy

The 3D printed scaffolds were characterized before and after plasma treatment using a 3D laser scanning microscope (KEYENCE, VK-X210, KEYENCE, Osaka, Japan). This captures both images of the surface and laser scans. Surface-specific values such as surface roughness (center roughness Sa) can then be measured, analogous to our previous work [11,14]. To investigate the surface roughness before and after plasma treatment of the scaffolds, images were acquired using the 3D laser microscope and the surface roughness was measured at four random locations. A scaffold was examined before plasma treatment and after plasma treatment.

##### Characterization by Immunoassay for Collagen I

To prove the successful coating of the scaffolds with collagen, immunofluorescence staining with an antibody against collagen I was performed. For this purpose, collagen-coated scaffolds and uncoated scaffolds were used as control group. The scaffolds were first washed with phosphate buffered saline (PBS). After washing, they were incubated for 45 min at room temperature in a so-called blocking solution. The blocking solution consisted of PBS, 1% BSA, and 0.1% Triton-x 100. Subsequently, the scaffolds were washed again 3 times with PBS. The primary antibody was then added, which was previously dissolved 1:1000 in a buffer (lowcross buffer). For the detection of collagen, the mouse anti-human collagen 1 (company info, ab6308) mouse antibody at a concentration of 1.5 mg/mL was used as the primary antibody. After addition of the primary antibody, the scaffolds were incubated at 4 °C overnight. Subsequently, the scaffolds were washed again 3 times with PBS. The scaffolds were then incubated in the secondary antibody. A goat anti-mouse Alexa 488 antibody (ThermoFisher Scientific) dissolved 1:500 in a lowcross buffer for 60 min at room temperature. These antibodies were labeled with a fluoreschrome, allowing them to be examined later with a fluorescence microscope (Olympus BX-53, 500 ms illumination time) (excitation at 495 nm (blue), emission 519 nm (green)). 

##### Characterization by ESEM

To study the mineralization of the collagen layer, this sample had to be prepared. A cryo-fracture had to be prepared. To do this, finished samples were held in liquid nitrogen at −196 °C for 30 s, following the procedures presented. The frozen samples were broken into two pieces using a 10 mm chisel. Breaking in the frozen state resulted in an optimal breaking edge. The resulting fracture edge also allowed a view into the coating. For this purpose, the edge areas of the strands oriented perpendicular to the fracture were viewed. Furthermore, otherwise only optically significant and deviating areas were examined more closely in order to record further information as well as anomalies. The measurement parameters in the FEI Quanta 250 FEG were: 20 kV accelerating voltage, the use of a Large Field Gaseous, detector (LFD) for secondary electrons, low vacuum of 130 Pa.

##### Characterization by MicroCT

Samples examined with the µCT must undergo several preparation steps before examination. First, they were cryo-fractured, comparable to the ESEM samples (see chapter before). Unlike the preparation of the ESEM images, however, this was not used to expose individual strands to allow insight into the coating, but merely to reduce the size of the sample. Sections of approximate size 1 mm × 1 mm × 2.04 mm were taken. The now cryo-broken sample pieces were thoroughly rinsed three times with PBS and then transferred to an ethanol series for dehydration. Here, all samples were transferred sequentially to 30%, 50%, 70%, 80%, 90%, 96%, and 100% ethanol for 30 min each. To fix the samples, the samples were transferred to a 24 well plate containing 1 mL/well (≥99.0%) hexamethyldisalazane for 4 h [22]. Finally, samples were removed from the hexamethyldisalazane and dried under a fume hood for 8 h. Dry samples were attached to the sample holder with superglue and then examined using µCT. The parameters used for the examinations with the µCT Skyscan 1272 system were as follows:

- Tube voltage: 40 kV
- Tube current: 250 µA
- Exposure time: 1815 ms
- Additional filtering: No additional filtering
- Binning: 1 × 1 (projection size: 4032 × 2688)
- Voxel size: 2.0 µm
- Rotation step: 0.15 degrees
- Frame averaging: 5
- 360° scan
- Random movement off

##### Characterization by TEM/EDX

For the TEM and EDX investigations, the PCL moldings were coated with collagen and treated with poly-L-aspartic acid. Subsequently, the specimens were cold-embedded in PELCO^®^ 10505 silicone embedding molds in Rencast FC52 (Goessl-Pfaff GmbH, Karlskron, Germany). Some samples were incubated in osmium (OsO4) for better detection of the collagen layer. Thin sections of the embedded samples were then prepared on the PowerTome XL ultramicrotome (Boeckeler Instruments Inc., Tucson, AZ, USA).

### 2.3. Statistics

Data are expressed as mean ± standard deviation of the mean and were analyzed by one-way analysis of variance (ANOVA). The level of statistical significance was set at *p* < 0.05. For statistical calculations, the Origin 2020 Professional SR1 (OriginLab, Northampton, MA, USA) was used.

## 3. Results

### 3.1. Characterization of the Scaffolds and Coatings

#### 3.1.1. Characterization by 3D Laser Scanning Microscopy

No significant difference in surface roughness was found between the two groups (without and with plasma treatment). The mid-surface roughness Sa for the non-plasma treated specimens was 4.19 + 0.22 µm and for the plasma treated specimens 4.70 + 0.79 µm. The length, height, pore size, and strand width were also measured by means of 3D Laserscanning microscopy (3D LSM) (pls see Table 4). The pore size was measured on the 3D LSM images by using ImageJ. However, there was a significant difference in surface roughness between uncoated (4.1 ± 0.1 µm) and coated scaffolds (3.35 ± 0.3 µm) with *p* < 0.05.

#### 3.1.2. Characterization of Collagen Coating by Immunoassay

**Figure 1:**
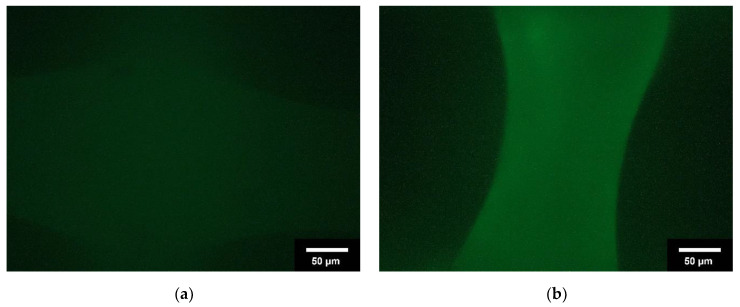
Overview of immunostaining; (**a**): uncoated scaffolds; (**b**): collagen-coated scaffolds. Images taken with Olympus BX-53 Fluorescence microscope @ 500 ms illumination time.

The immunoassay showed that a collagen layer had formed on the scaffolds. In the following Figure 1, the intrinsic fluorescence of the unloaded scaffold is shown in (a) and the fluorescence of the loaded scaffolds is shown in (b). It is evident that the intrinsic fluorescence is very weak, whereas the fluorescence of the samples is very pronounced depending on the thickness of the collagen layer.

#### 3.1.3. Characterization by Means of ESEM

##### Classical Collagen Coating

**Figure 2:**
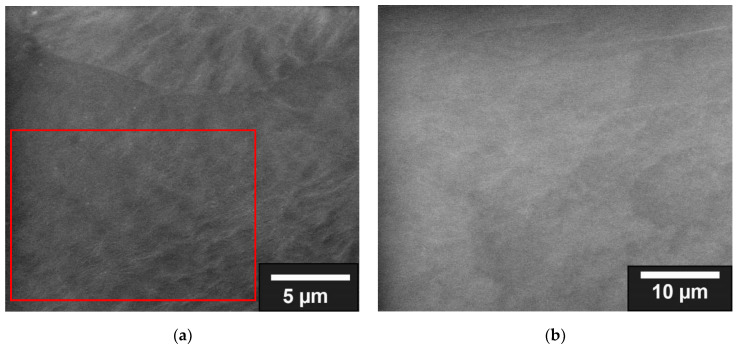

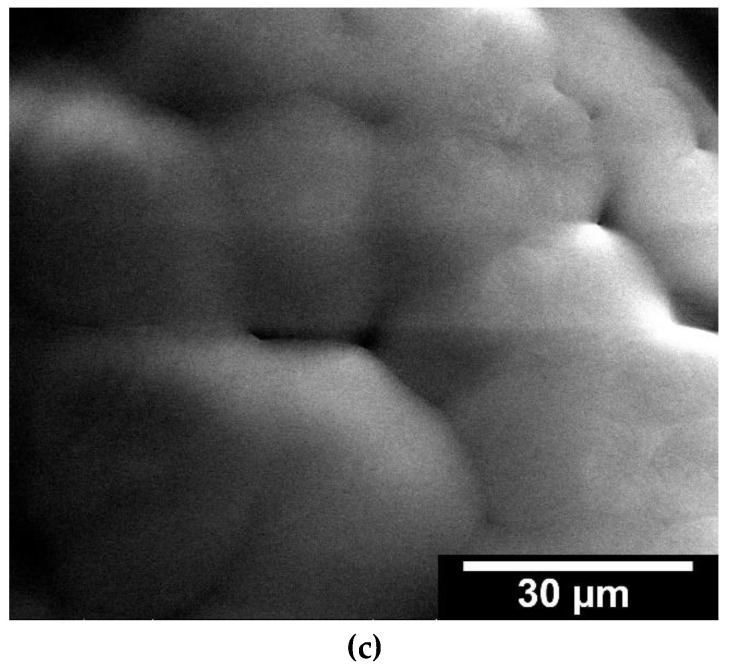
ESEM images of collagen coating by incubation; the red area in (**a**) is shown enlarged in (**b**); the uncoated scaffold is shown in (**c**).

In the classical form of collagen coating, incubation in the collagen solution, collagen was deposited homogeneously on the surface of the scaffolds (see Figure 2). The thickness of the layer depended on the incubation time. In addition, it can be seen in Figure 2 that the uncoated scaffolds have gaps between the fused PCL particles, which were closed in a and b by the coating.

##### Collagen-HA Coating

**Figure 3:**
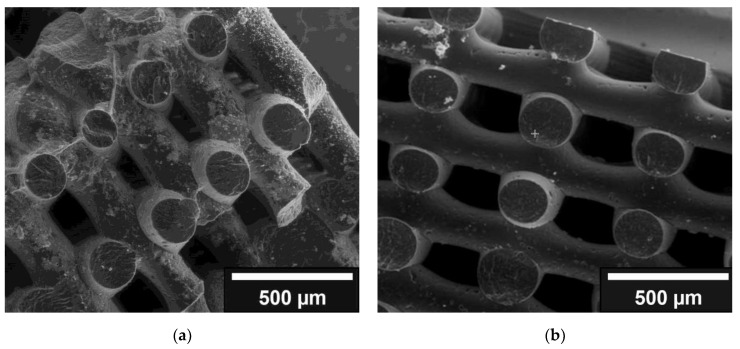
Comparison of collagen-HA coatings: (**a**): gelled collagen-HA coating; (**b**): collagen-HA coating, which has been diluted with acetic acid.

Due to the change in pH from slightly acidic to basic, the first mixing of collagen solution with nano HA resulted in the gelation of the suspension. To avoid this, all further tests were carried out with the addition of acetic acid. Figure 3 shows the inhomogeneous coating with gelled collagen HA coating. This is contrast with the collagen HA coating diluted with acetic acid. However, the dilution step changed the concentration of collagen + HA, which is reflected in a less effective coating.

##### Surface Coating by Incubation in 10× SBF

**Figure 4:**
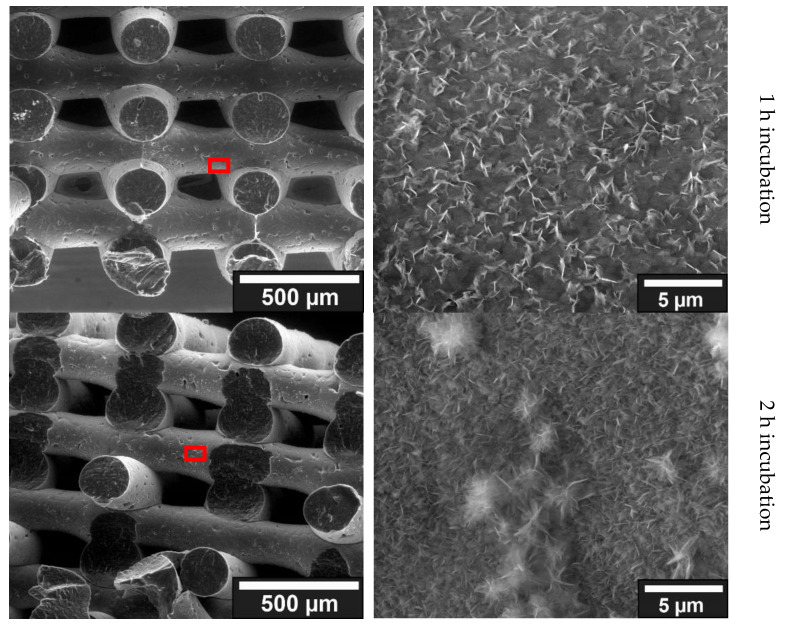

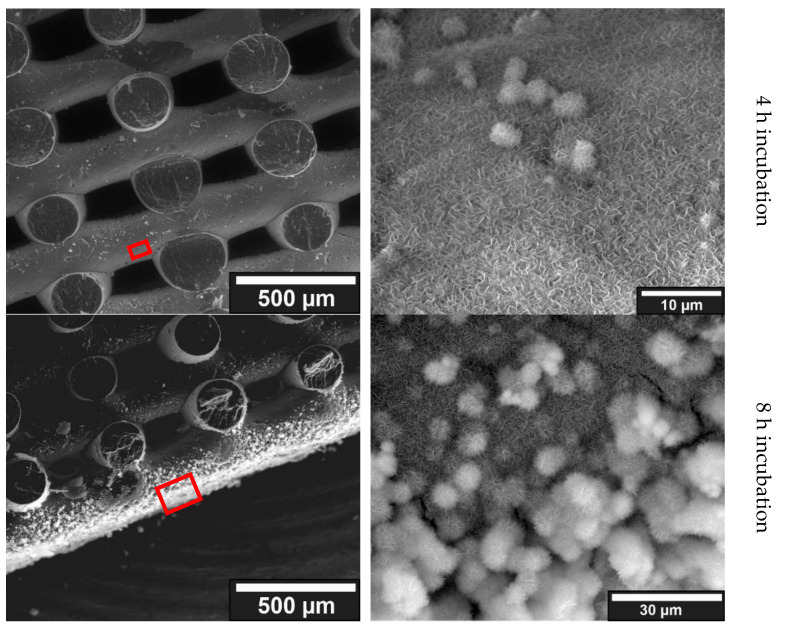
Comparative ESEM images of the coatings formed by incubation in 10× SBF. Incubation time varies from top to bottom: 1, 2, 4, and 8 h. The left image always shows the scaffold at 188× magnification, and the right image shows the crystalline structures and agglomerates on the surface. Because the crystalline structures and agglomerates increase over the incubation time, the magnification factor also varies. ESEM images were taken with a FEI Quanta FEG 250 @ 20 kV, 130 Pa, Large Field Detector.

The results of storage in 10× SBF for one hour showed no major agglomerations of crystalline structures, see overview image (Figure 4). However, a surface coating can already be recognized by the lighter shimmering. Looking at the individual results, crystalline structures are already clearly visible both in the analysis of the fracture edge and on the surface of a strand. In the top view of the fracture edge, the ridge line of protruding crystalline structures can also be seen. It is also clear that there is a lighter area between the ridge line and the PCL. This indicated that there is a layer between the PCL and the protruding crystalline structures. This is probably the collagen layer. When looking at the open area of a strand, crystalline structures were also visible, which are partially agglomerated. The results of the 2 h samples showed few changes compared to the samples immersed in 10× SBF for 1 h. In general, only an increased agglomeration of the crystalline structures already found in the samples of the 1 h storage is evident, this can also be seen in the overview images (Figure 4; left image). When looking at the fracture edge and the surface of a strand of the first sample, agglomerations of crystalline structures can be seen. On closer inspection of the second sample of the 2 h storage, clearly larger crystalline structures can be seen in the observation of the fracture edge; however, these could be related to the generally larger agglomerations that can be observed in the 2 h samples on the open strand. The results of the 4 h samples also showed comparable results with those of the 1 h and 2 h samples. The observed crystalline structures are larger and more defined compared to the 1 h and 2 h samples. Furthermore, the observed agglomerations of crystalline structures were also larger. Uncoated areas can be observed in both samples. A closer look at the fracture edge of the first sample again revealed crystalline structures on the surface. A top view of the open face of one strand showed an increased density and size of crystalline structures as well as some agglomerated structures. The samples that were incubated for 8 h in 10× SBF showed the largest agglomerations and thickest crystalline structures. Further magnification of both samples clearly showed, compared to the previous samples, even more defined and thicker crystalline structures as well as larger agglomerations in places. Thus, also when looking into the coating, much larger crystalline structures can be seen in both samples.

##### Mineralization with ALP

**Figure 5:**
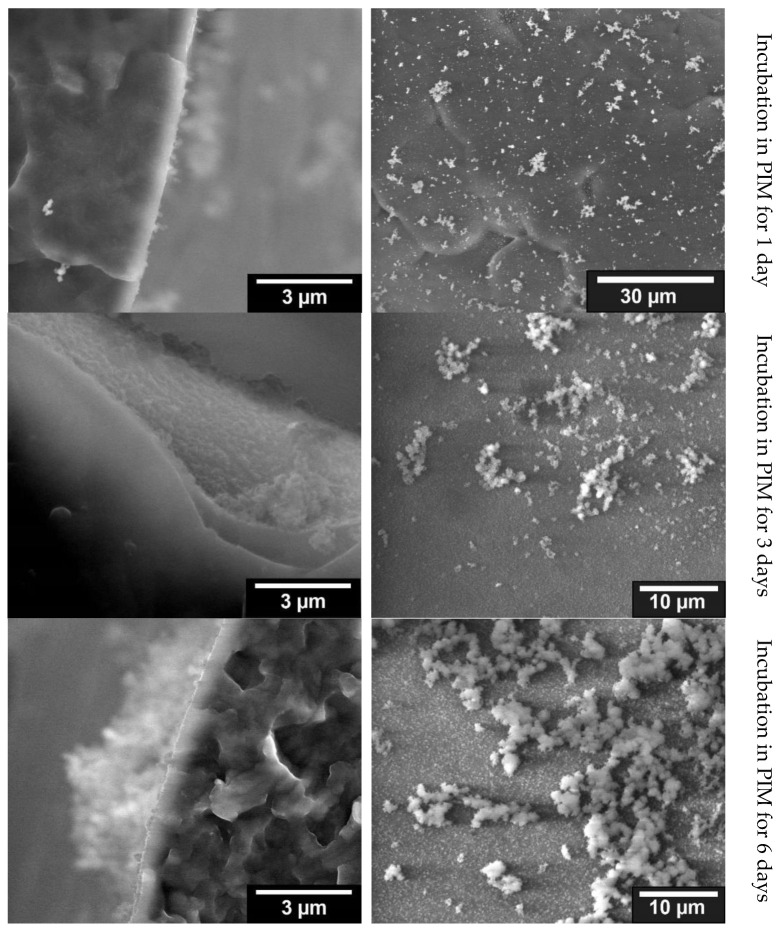
Comparison of the ESEM images for the different incubation times in PIM; (**left**): fracture edge of a cylindrical strand with coating on the outer surface; (**right**): surface of a strand with agglomerations of the crystalline structures.

The samples incubated for one day in PIM activated with ALP already showed first agglomerations of crystalline structures. Among and next to these agglomerated structures, crystals can also be found shining through as small dots on the ESEM images. The presence of small crystalline structures as well as agglomerations of these crystallites resembled, on a smaller scale, the results of the 10× SBF experiments. The samples incubated in PIM for 3 days continued to show crystalline structures under ESEM both in the observation of the fracture edge and on the surface of a strand. Compared to the 1-day sample, a finer distribution of small crystallites as well as more agglomeration were evident when looking at the surface. Looking at the fracture edge also showed a thicker surface layer compared to the 1-day sample. The images of the 6-day sample were comparable to the results observed in the 1-day and 3-day samples. The same small crystals as well as agglomerations were found when looking at the open area of a strand (blue). Again, the agglomeration increased with longer stored samples. However, the size of the observed crystals did not increase with longer storage. Observation of the fracture edge also revealed a larger layer of crystals on top of those protruding from the surface coating. When comparing the three different samples, the longer the storage in the ALP activated PIM, the stronger the agglomeration. This can be observed particularly well on the surface due to the occurrence of larger agglomerations (see Figure 5).

##### Mineralization with Poly-L-Aspartic Acid

**Figure 6:**
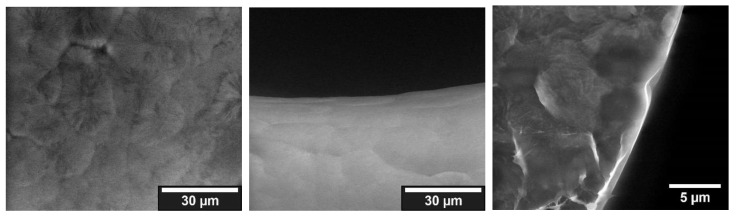
ESEM images of fracture edge (**left**) and surface of a strand (**center**), and magnification of the surface (**right**) of a sample treated with poly-L-aspartic acid.

The samples treated with poly-L-aspartic acid showed no observable crystals under ESEM, and the surfaces appeared untreated except for a discernible collagen layer. The observable collagen layer through which the PCL shines through showed no signs of mineralization (cf. Figure 6). The uniformity of the coatings and the thickness of the coatings were summarized in Table 5.

#### 3.1.4. Characterization by MicroCT

The investigations using µCT were only carried out for the ALP and poly-L-aspartic acid coatings.

##### Coatings with ALP

**Figure 7:**
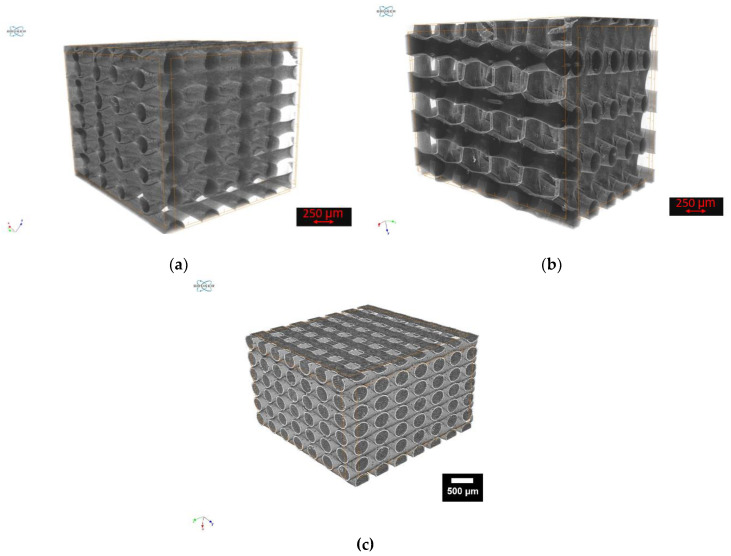
MicroCT images of samples incubated in: (**a**): ALP-activated PIM; (**b**): poly-ASP; (**c**): uncoated sample.

The results of the µCT examinations showed the presence of a surface coating on the ALP samples, but no crystals can be observed within the collagen layer. The surface coating is most evident when comparing the top and bottom surfaces of the constructs. Since the bottom side rested on the bottom of the corrugated sheet due to gravity during the coating with collagen and during storage in the ALP activated PIM, no surface coating could occur on the bottom side (cf Figure 7a).

##### Coatings with Poly-L-Aspartic Acid

The results of the µCT examinations of the poly-ASP samples showed, as already the examinations of the ALP samples, a surface coating. However, no crystalline structures were visible here either (cf. Figure 7b) compared to the uncoated sample (cf. Figure 7c).

#### 3.1.5. Characterization by TEM/EDX

**Figure 8:**
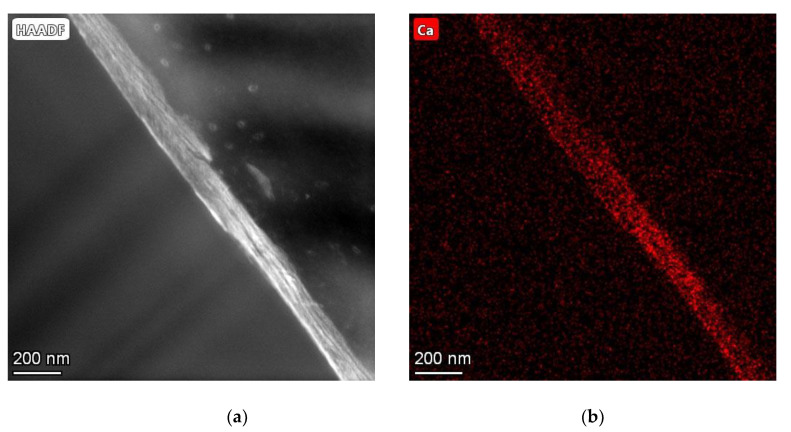
TEM (**a**) and EDX (**b**) images of thin sections of collagen-coated PCL, post-treated with poly-L-aspartic acid; TEM image taken with TALOS 200X, 185,000× magnification, HFW 546 nm, STEM HAADF, EDX measuring with FEI SuperX EDX System.

Within the thin sections of the coated samples, HA nanocrystals have been detected in the collagen layer by TEM and subsequent EDX. The detection was done via EDX of Ca; this is neither contained in PCL nor in collagen (see Figure 8).

## 4. Discussion

Collagen coating in the present project was performed analogously to work we have already published [11,14]. 

### 4.1. Collagen-HA Coatings

The addition of 5 wt% HA as a nanopowder did not result in a homogeneous surface coating consisting of collagen and HA. The HA did not distribute homogeneously and dried out and agglomerated on the surface of the scaffold. However, non-agglomerated homogeneous distribution of HA has been successful in other work [23]. This raises the question of why this was not successful in this work. The failure of homogeneous surface coating was due to the phase separation of the resulting collagen-HA gel. A possible gel formation had not been considered. Subsequently, a reference to this gel formation was found in the work of Oechsle et al. [24]. In this case, after gelation, the solid phase, the HA, separated and dried out in the subsequent step inhomogeneously on the surface. This gel formation was related to the pH, which could be confirmed by dissolving the gel when acetic acid was added. When the pH was changed, initiated by the addition of the HA nanopowder, electrostatic binding of the collagen molecule occurred, a phenomenon known for example with chitsoan molecules [25]. Since collagen behaves similarly to chitosan, this could be the reason for the unexpected gelation.

In the second set of experiments, in which only 2 wt% HA was added, similar gel formation was seen. Accordingly, the amount of HA added does not seem to be relevant to the formation of the gel. Consequently, the gel was dissolved by adding 0.2 M acetic acid. This 0.2 M acetic acid with a pH of 3.5 corresponds to the solvent of the commercially purchased collagen solution from Corning. The addition of the acetic acid lowered the pH, which dissolved the gel. This confirms that gelation is strongly linked to pH [25]. Twice the amount of acetic acid was needed to dissolve the gelation than collagen solution originally used. This, of course, also reduced the concentration of dissolved collagen and HA, and since 600 μL per scaffold continued to be added, insufficient collagen could be deposited on the surface, which is especially evident when comparing the ESEM results of the two sets of experiments. Working with pure collagen and other solvents may well lead to the desired HA/collagen coatings as demonstrated by the AG of Yeo et al. [23].

### 4.2. Incubation in 10× SBF

The experiments on the storage of collagen-coated scaffolds in 10× SBF showed that it was possible to obtain a homogeneous coating of HA on the collagen. The crystals and agglomerations produced in this regard are very similar to the HA crystals observed in the studies of Vaquette et al. [17], Poh et al. [16], and Yang et al. [19]. Furthermore, the expression and size of the crystals as well as the size of the agglomerations of the crystals could also be modulated by prolonged storage, a result that was also observed in the work of Yang et al. [19]. Furthermore, similar to the observations of Yang et al. [19] and Tas et al. [26], using 10× SBF for the preparation of a HA layer shortened the time to form this layer enormously, compared to normal SBF (i.e., 1× SBF without tenfold increased ion concentration). Thus, a very rapid formation of crystalline structures (within a few hours after storage) was also observable in the context of this work, which is a further an indication that the crystals formed are HA.

### 4.3. Coatings with ALP

The samples from the ALP experiments also showed successful surface coating with HA. Crystalline structures can be seen on the surface. These behaved similarly to the previously observed crystals of the 10× SBF samples. Thus, isolated crystals and agglomerations of different sizes are shown (Figure 4 and Figure 5). These observed crystals are also optically similar to the HA crystals observed in the work of Jaroszewicz et al. [20].

### 4.4. Coatings with Poly ASP

The samples of the PolyASP experiments also showed a well recognizable collagen layer on the surface. This is further confirmed by the µCT images as well as the TEM + EDX images. Here, a surface coating can be seen on all images, which is different from the PCL. However, there were no crystalline structures on any of the images that indicate the presence of HA. In the work of Deshpande et al. [21], however, collagen was shown to mineralize successfully. The investigation with the TEM and EDX could prove the mineralization of the collagen. As mentioned earlier, the HA crystals observed by Deshpande et al. [21] were only a few 50–100 nm in size and were present in a layer above the collagen layer. This requires not only a view into the collagen layer (which was attempted with TEM + EDX and the ultra-thin sections required for this), but also the magnification required at the nanometer scale. This could be confirmed by our measurements. Calcium and oxygen were detected within the collagen layer by EDX, where collagen contains no calcium atoms. This would also correspond to the in vivo occurrence of HA in the form of crystals with a size of a few nanometers, which Deshpande et al. [21] tried to emulate with their method.

#### Implications with Respect to Application

Coating with HA crystals in collagen solution have proven to be impractical as the HA clumped in the solution and could not be applied homogeneously. For simple surface mineralization of collagen coatings but also simply for deposition of nanocrystalline HA on smooth surfaces, both incubation in SBF and ALP are suitable, with 10× SBF being the cheaper option with which thick HA layers can be generated in a short time. However, if the HA nanocrystals are to be present in the coating itself (similar to bone), the coating method with incubation in poly-ASP should be used. There is, of course, still a need for optimization with regard to the quantity and orientation (with regard to bones from the petri dish) of the HA nanocrystals within the collagen layer.

## 5. Conclusions

In the present work, we examined a wide variety of approaches to determine whether and where HA layers form on collagen-coated PCL scaffolds. The HA layers formed in various forms only at (not in) the collagen layer for all methods except incubation in poly ASP. However, through poly ASP treatment, nanocrystalline HA could also be detected within the collagen layer.

## Figures and Tables

**Table 1:** Parameters for the 3D printing of PCL.

Used Needle	Pressure	Temperature	Speed	Needle-Offset	Pre/Post Flow	Temperature Underground
24G	4–5 Bar	80 °C	1.0 mm/s	0.19 mm	0.07 s pre 0.10 s post	17 °C

**Table 2:** Order and amount of substances required for 10× SBF.

Order	Substance	Amount (for 1 L 10× SBF)
1	NaCl	58.430 g
2	KCl	0.373 g
3	CaCl_2_–2 H_2_O	3.675 g
4	MgCl_2_–6 H_2_O	1.016 g
5	Na_2_HPO_4_–H_2_O	1.633 g

**Table 3:** Sequence and amount of substances necessary for PIM.

Sequence	Substance	Amount (for 45 mL PIM)
1	TRIS buffer	545.13 mg
2	C_3_H_7_Na_2_O_6_P 5 H_2_O	300 mg
3	CaCl_2_	200 mg
4	MgSO_4_	50 mg
5	NaN_3_	9 mg

**Table 4:** Dimensions of the 3D-printed scaffolds.

Parameter	PCL Scaffold
Length (mm)	8.42 ± 0.01
Height (mm)	2.04 ± 0.03
Pore size (µm)	295.4 ± 9.8
Strand width (µm)	300 ± 12.6
Porosity (%)	31.9

**Table 5:** Comparison of the coating methods with regard to uniformity of the HA coating and thickness of the coating.

Coating Method	Uniformity of Coating	HA Coating Thickness (µm)
Collagen-HA	non-uniform coating, HA already clumps in the collagen solution, HA only on the collagen coating, not within	-
SBF (10×)	depending on the incubation time, short incubation (1 h) leads to uniform coating; moreover, formation of a uniform nanocrystalline layer with spots on the surface, whose expression increases with time, HA only on the collagen coating, not within	1 h: <1 µm 2 h: 1–3 µm 4 h: 3–6 µm 8 h: 10–30 µm
ALP	uniform coating, no nanocrystalline HA, as incubation time progresses, increased appearance of agglomerates on the surface, whose size and density increase with time, HA only on the collagen coating, not within	1 d: <1µm 3 d: 1–2 µm 6 d: 2–4 µm
PolyASP	HA only detectable by high-res EDX within the collagen layer, no HA nanocrystals detectable at the outer collagen layer	-

## Data Availability

The data presented in this study are available on request from the corresponding author.
